# Neural Plasticity in Mood Disorders

**DOI:** 10.1155/2018/3745251

**Published:** 2018-05-09

**Authors:** F. Scott Hall, Aijun Li, Bingjin Li

**Affiliations:** ^1^Department of Pharmacology and Experimental Therapeutics, University of Toledo College of Pharmacy and Pharmaceutical Sciences, Toledo, OH, USA; ^2^Department of Integrative Physiology and Neuroscience, Washington State University, Pullman, WA, USA; ^3^Jilin Provincial Key Laboratory on Molecular and Chemical Genetics, Second Hospital, Jilin University, Changchun, Jilin, China

The contribution of differences in cellular and anatomical function to mood disorders has been a focus of research attention in biological psychiatry for a very long time. Some of the earliest structural imaging studies demonstrated differences in the size of certain brain structures in major depression, bipolar disorder, posttraumatic stress disorder, and drug dependence [1–4]. This led to the search for the cellular bases of these changes and how genetic and environmental factors contribute to these differences [5]. This is, of course, a topic of much ongoing research that seeks to identify these mechanisms at a cellular and anatomical level, as well as to relate these changes to both internal and external causal factors. An understanding of these factors will not only provide an understanding of the aetiology of affective disorders but will also inform efforts to develop new treatments for these conditions.

The contributions to this special issue address many aspects of this still-developing field, including summarizing changes in neural plasticity in brain regions important in depression, including the hippocampus, amygdala, and prefrontal cortex (W. Liu et al., “The Role of Neural Plasticity in Depression: From Hippocampus to Prefrontal Cortex”). New findings indicate that there are abnormalities in the functional connectivity of the anterior insula in depressed asthmatic patients (Y. Zhang et al., “Abnormal Functional Connectivity of Ventral Anterior Insula in Asthmatic Patients with Depression”). Differences in functional connectivity may influence brain activity involved in emotional states, as summarized in another review (R. Ding et al., “Emotion Processing by ERP Combined with Development and Plasticity”). Hypoxic injury associated with a variety of conditions has also been shown to contribute to the development of depression (F. Zhao et al., “Effect of Hypoxic Injury in Mood Disorder”), as summarized in another review, and in addition to hypoxic injury to neurons, such injury also affects neuroplasticity in many of these same brain regions.

As our understanding of the mechanisms underlying (generally) reduced neuroplasticity in many brain regions in depression evolves, efforts to normalize neural plasticity in depression continue to develop. Many of these new treatments, as well as a developing understanding of the mechanisms underlying the effects of older treatments, suggest that alterations in glutamatergic mediated neuroplasticity are critical for their antidepressant actions, including for the so-called “fast-acting” antidepressants (Y.-J. Huang et al., “New Treatment Strategies of Depression: Based on Mechanisms Related to Neuroplasticity”). A new report in this volume suggests that Yueju, a Chinese traditional medicine, not only has antidepressant effects in the learned helplessness model but also involves similar mechanisms to other antidepressants (e.g., PKA, CREB, BDNF, and NMDA receptors) (Z. Zou et al., “Neural Plasticity Associated with Hippocampal PKA-CREB and NMDA Signaling Is Involved in the Antidepressant Effect of Repeated Low Dose of Yueju Pill on Chronic Mouse Model of Learned Helplessness”). The effects of Yueju were greater than fluoxetine, and a previous report indicates that Yueju may be a fast-acting antidepressant [6]. Several other papers in this special issue address other understudied mechanisms that appear to contribute to neuroplasticity in mood disorders and related conditions. These include the importance of zinc and how zinc might contribute to antidepressant actions through effects on monoaminergic systems (U. Doboszewska et al., “Zinc in the Monoaminergic Theory of Depression: Its Relationship to Neural Plasticity”) and the role of histidine triad nucleotide-binding protein (P. Liu et al., “HINT1 in Neuropsychiatric Diseases: A Potential Neuroplastic Mediator”).

Although most treatments for mood disorders emphasize pharmacological treatments, nonpharmacological treatments are of course important as well, particularly in combination with pharmacotherapy. As discussed in two reviews here, physical activity produces antidepressant-like effects that are mediated by differences in brain plasticity (C. Phillips, “Physical Activity Modulates Common Neuroplasticity Substrates in Major Depressive and Bipolar Disorder”), and these effects work through similar mechanisms to pharmacotherapies such as brain-derived neurotrophic factor (BDNF) (C. Phillips, “Brain-Derived Neurotrophic Factor, Depression, and Physical Activity: Making the Neuroplastic Connection”). Sleep is well known to be disrupted in depression, and sleep disturbances disrupt a number of neuroplastic mechanisms that are important for memory formation and other processes (M.-Q. Zhang et al., “Neural Plasticity Is Involved in Physiological Sleep, Depressive Sleep Disturbances, and Antidepressant Treatments”). These authors go on to consider the mechanisms underlying therapeutic sleep deprivation, which appears to involve at least some of the same mechanisms as other antidepressant treatments, including normalizing some aspects of neural plasticity that are disrupted in depression.

The link between depression and chronic pain is well known epidemiologically, but increased understanding of both neuropathic pain and the mechanisms of depressive disorders has identified substantial overlap in underlying mechanisms (J. Sheng et al., “The Link between Depression and Chronic Pain: Neural Mechanisms in the Brain”). As discussed in this review, these mechanisms involve surprisingly similar alterations in glutamatergic synapses and similarities in the driving factors of these neuroplastic changes, including monoamines, BDNF, and inflammatory mechanisms. As synthetic opioids have become widely used for chronic pain, as well as widely abused, this raises important questions about the role of opioids in depression, the potential antidepressant actions of acute opioid treatments, and the potential for chronic opioid treatments to induce depression as a consequence of tolerance and withdrawal. The incidence of addiction is, of course, increased in patients with various pain conditions and mood disorders. Hypotheses about the causes of increased addiction in mood disorder patients include self-treatment for underlying mood and cognitive dysfunction [7], as well as more direct effects on drug actions or drug-related phenotypes. As discussed in a review in this volume, genome-wide association studies for drug dependence and genetic studies in mice have found that genetic variation or modifications of cell adhesion molecule genes may be a major contributor to addiction liability (D. E. Muskiewicz et al., “The Role of Cell Adhesion Molecule Genes Regulating Neuroplasticity in Addiction”). This indicates that there is a fundamentally important role for neural plasticity in addiction and in particular in the genetic liability for addiction. It remains to be seen to what extent this genetic contribution to addiction liability overlaps with genetic contributions to mood disorders.

Collectively, the articles presented in this special issue represent several novel research directions that are contributing to our understanding of the importance of neural plasticity in mood disorders and related conditions. This understanding will help lead to improved pharmacological and nonpharmacological treatments for these conditions.

*F. Scott Hall* *Aijun Li* *Bingjin Li*
